# Fermenting Bread Dough as a Cheap, Effective, Nontoxic, and Generic Attractant for Pest Snails and Slugs

**DOI:** 10.3390/insects12040328

**Published:** 2021-04-07

**Authors:** Robin Veasey, Maria Cordoba, Andrew Colton, Leonard Fujimoto, Christine Dodge, Ian Foley, Gary Adams, Taelor Anderson, Richard Merenz, Arnold Hara, Amy Roda, Jocelyn Millar, Rory Mc Donnell

**Affiliations:** 1 Department of Entomology, University of California, Riverside, CA 92521, USA; robin.veasey@ucr.edu (R.V.); jocelyn.millar@ucr.edu (J.M.); 2 Department of Crop and Soil Science, Oregon State University, Corvallis, OR 97331, USA; macordot@gmail.com (M.C.); andrew.colton@oregonstate.edu (A.C.); christine.dodge@oregonstate.edu (C.D.); 3 Department of Plant and Environmental Protection Services, University of Hawaii, Hilo, HI 96720, USA; lennyhf@hawaii.edu (L.F.); arnold@hawaii.edu (A.H.); 4 Montana Department of Agriculture, University or Institute, Helena, MT 59601, USA; IFoley@mt.gov; 5 USDA-APHIS-PPQ, Billings, MT 59102, USA; gary.d.adams@usda.gov (G.A.); taelor.o.anderson@usda.gov (T.A.); joemerenz@gmail.com (R.M.); 6 Animal and Plant Health Inspection Service, Plant Protection and Quarantine, United States Department of Agriculture, Miami, FL 33158, USA; amy.l.roda@usda.gov

**Keywords:** terrestrial gastropods, slugs, snails, invasive species, pest management, bread dough, fermentation, lure

## Abstract

**Simple Summary:**

Snails and slugs are key pests of crops but control options are limited with an overreliance on molluscicides, which have variable efficacy. Thus, there is an urgent need to improve the performance of these pesticides, and one option is to identify more efficacious attractants for incorporation into baits and/or use in traps. Our results showed that a simple bread dough (flour, water, and yeast) was highly attractive to six invasive pest gastropod species in both laboratory and field trials in Hawaii, Oregon, and Montana. The dough remained attractive for at least 8 days and was significantly more attractive than a widely used toxic bait (Deadline^®^ M-Ps™). Given its simplicity, low cost, and the ready availability of its ingredients, the dough has potential to be used in developing countries where access to commercial molluscicides is limited by cost. In addition, a premixed dry formulation of flour and yeast, where water simply needs to be added to activate the bait, would likely have an indefinite shelf life and be readily shippable, both highly desirable properties for an operational lure. Thus, bread dough constitutes a nontoxic, generic, and effective tool that could be used in the detection and management of pest gastropods worldwide.

**Abstract:**

Invasive slugs and snails are among the most damaging pests of agriculture in temperate and tropical regions of the world. Control options, however, are limited and there is a heavy reliance on chemical molluscicides of variable efficacy. There is an ongoing need to improve management methods. Here, we show that a simple fermenting bread dough formulation (flour, water, and yeast) was effective in attracting pest mollusk species in laboratory tests, and in multiple replicated field trials in Hawaii, Oregon, and Montana. The dough attracted substantially more terrestrial pest gastropods, including invasive species of major economic importance such as *Cornu aspersum*, *Deroceras reticulatum*, *Ambigolimax valentianus*, *Xerolenta obvia*, *Lissachatina fulica*, and *Parmarion martensi*, than water controls. The dough remained attractive for at least 8 days and was significantly more attractive than a widely used metaldehyde-based bait, Deadline^®^ M-Ps™. Thus, fermenting bread dough represents a nontoxic, generic, and effective tool to aid in managing pest gastropod infestations, either using baited traps or in attract-and-kill approaches. Given its simplicity, low cost, and the ready availability of its ingredients, the dough also has potential to be used in developing countries where access to commercial molluscicide baits is limited by cost.

## 1. Introduction

Snails and slugs (Mollusca: Gastropoda) are among the most significant and intractable pests of agriculture, and crop losses due to these invertebrates have been reported throughout recorded history [1]. With the dawn of the 20th century, gastropods emerged as significant pests in temperate and tropical regions largely due to the intensification of agriculture, development of new crops, purposeful introductions [2], and particularly for the last several decades, spread through increases in global trade [1]. However, control measures for these pests are limited, with an overreliance on the use of chemical molluscicides [3,4,5]. Furthermore, the performance of existing bait formulations is highly variable; for example, single applications of molluscicidal baits typically achieve only 10 to 60% slug mortality [6,7]. Alternatively, although biological control of pest slugs using the nematode *Phasmarhabditis hermaphrodita* has been used successfully by growers throughout Europe, the high cost of the product (Nemaslug^®^) is a distinct disadvantage in comparison to molluscicides [8]. Likewise, barriers including copper tape have been used to help manage these pests, but due to their expense, they tend to be used primarily in high value crops [9]. Gastropod management problems have been exacerbated in recent years by an increase in adoption of no-till and conservation tillage by farmers. Such practices preserve crop residues and soil moisture, but simultaneously create an environment suitable for gastropod proliferation [10]. Slug and snail species continue to invade new regions of the world [11,12,13,14,15] and crop damage by invasive gastropods will likely increase with climate change [16]. Furthermore, many gastropod species serve as intermediate hosts of the causative agents of potentially lethal diseases, such as the rat lungworm, *Angiostrongylus cantonensis*, which causes angiostrongyliasis and eosinophilic meningoencephalitis in humans [17]. For all these reasons, there is an ongoing need to improve the effectiveness of gastropod management strategies. One such approach is to identify more efficacious attractants for incorporation into existing baits and/or use in mass trapping programs.

In comparison to pest insects, the chemical ecology of snails and slugs remains largely unexplored, especially with regard to exploiting the olfactory cues that these animals use to locate food, or possible chemical signals that may regulate intraspecific communication [18,19,20]. Although beer has long been recommended as a gastropod attractant [21,22], other fermenting products have largely been overlooked, despite their use in management of some insect pests [23]. This provided the incentive for the study reported here, in which the attractiveness of fermenting bread dough was assessed against a broad range of species, including both temperate (the grey field slug *Deroceras reticulatum*, the European brown garden snail *Cornu aspersum*, the three-band garden slug *Ambigolimax valentianus*, the heath snail *Xerolenta obvia*) and tropical (giant African land snail *Lissachatina fulica*, the semislug *Parmarion martensi*) invasive pest species.

*Deroceras reticulatum* has a global distribution and is one of the most important invasive slug species worldwide. It causes serious damage above and below ground to a wide range of crops [3,24]. *Ambigolimax valentianus* is a pest of ornamental plants [6,25]. *Cornu aspersum* is a well-known pest of citrus [9], vineyards [26], and ornamentals [27]. *Lissachatina fulica* is considered among the world’s most damaging invasive pests, because it feeds on a wide variety of agricultural crops and native plants [28] and it represents a threat to human health by acting as an intermediate host of the nematode *A. cantonensis* which can cause the potentially fatal disease eosinophilic meningoencephalitis [29]. *Parmarion martensi* is a serious pest in Hawaii and other Pacific islands [30,31] and is also a known vector of *A. cantonensis* [17,30]. *Xerolenta obvia* has been reported as a pest of fodder crops and is a serious contaminant pest of fruits and vegetables in Europe. It has also been shown to vector fungal pathogens of crops and parasites of domestic animals [6].

The goal of this study was to test the hypothesis that terrestrial gastropods, which often feed on decaying organic matter [32], are attracted to fermenting baits. Our specific objectives were: (1) To test the attractiveness of fermenting bread dough to *C. aspersum* in laboratory bioassays; (2) to compare the attractiveness of fermenting bread dough with a widely used metaldehyde-based bait (Deadline^®^ M-Ps™) in laboratory choice tests; (3) to assess the performance of the bread dough under field conditions in Oregon, Hawaii, and Montana against the invasive gastropod species described above; and (4) to investigate the effect of aging on dough performance in the field.

## 2. Materials and Methods

### 2.1. Dough Formulation

In all of the laboratory and field bioassays the dough mixture was prepared from 500 mL of All Purpose Enriched Kröger^®^ brand bleached flour, 500 mL of water (deionized for laboratory tests and bottled water for field tests), and two packets (0.25 oz) of Red Star^®^ Active Dry Yeast from a local grocery store. All ingredients were mixed thoroughly in a large bowl to allow room for expansion, then held at ambient temperature until used in bioassays.

### 2.2. Laboratory Choice Bioassays with Cornu aspersum to Test the Attractiveness of Bread Dough

#### 2.2.1. Attractiveness of Dough of Various Ages

Using a high throughput methodology [18], the attractiveness of fermenting bread dough of various ages was investigated in a set of 5.08 cm diam glass T-tubes, using adult *C. aspersum* from a laboratory colony. Colonies were held in plastic containers (35.9 cm × 20 cm × 12.4 cm) with 10 snails per container and maintained in a growth chamber (Thermo Scientific Precision Model 818, Thermo Fisher Scientific Inc., Waltham, MA, USA) at 18 °C and 12 h photoperiod. The containers were kept moist with a single paper towel (Bounty Select-a-Size) saturated with deionized water. Specimens were fed organically grown carrots, with oyster shell powder supplied as a source of calcium. Towels, carrots, and shell powder were replaced three times weekly.

The dough was prepared as outlined above and allowed to age under ambient conditions in the laboratory. The attractiveness of the fermenting dough was tested daily for the first six days and then every other day until day 10. The dough (5 mL) was placed into a 15 mL plastic disposable portion cup (Crystal Ware Clear Portion Cups, Conlon Products, Inc., Lawrence, MA, USA). The same volume of deionized water was used as a control, in case the snails were simply attracted by humidity. The cups were placed at the ends of the arms of each T-tube. After placing the cups in position, an adult snail, which had been starved for 24 h, was placed in the stem of the T-tube. The snail was observed for 30 min, and when it reached a point 75% of the way up either arm of the T, it was considered to have made a choice [18], and the choice was recorded. Snails that failed to make a choice within 30 min were counted as non-responders. Snails were tested only once. After each replicate the T-tube was washed, the positions of the treatment and control cups were switched to account for any side preference and a new adult snail was added to the T-tube stem. Fifteen replicates were conducted for each age of dough. Bioassays were carried out under ambient lighting (12 h light: 12 h dark) and temperature (~22 °C) conditions in a windowless laboratory, between ~10:00 a.m. and 4 p.m.

#### 2.2.2. Attractiveness of Dough Aged for 2 Days versus Deadline^®^ M-Ps™

The same methodology was used as described above except that the relative attractiveness of 2-day old dough, which was shown to be highly attractive in Section 2.2.1, and Deadline^®^ M-Ps™ (AMVAC^®^ Chemical Corporation, Newport Beach, CA, USA) were compared. The latter is a widely used bait comprising inert ingredients, primarily a proprietary attractant, and metaldehyde (4%). Doses of 2.5 g of 2-day old dough, or the commercial bait were placed into separate 15 mL plastic disposable portion cups, which were placed at the ends of the arms of each T-tube. Twenty replicates were completed.

### 2.3. Field Trials with Dough Aged for 2 Days

Field sites were chosen based on the known presence of particular target species. The specific design and conditions for field bioassays with each species were modified to accommodate factors such as the size of the test species, location of gastropod populations within the field sites, and the logistics of working at sites remote from the laboratory, as described in detail below. The bread dough used in all field trials was 2-days old. 

#### 2.3.1. Field Trials with *Cornu aspersum*, *Ambigolimax valentianus*, and *Deroceras reticulatum*

Dough and water controls were deployed in Snailer^®^ traps (American Organic Products, Ventura, CA, USA), which are green, capsule-shaped, plastic traps with a one-way slatted door on each side and a top to keep out rain and debris (Figure S1). The bottom of each trap contained 150 mL of liquid metaldehyde (SlugFest^®^; OR-CAL, Inc., Junction City, OR, USA) solution at the labelled rate (30 mL in 38 L of water). Fifteen traps were baited with 10 mL of the bread dough in a 15 mL disposable portion cup (see above), with 15 additional traps with 10 mL of deionized water to control for the possibility of snails being attracted just by the humidity plume released from the dough formulation. Traps were set up daily just before sunset, when gastropods typically became active in the field. The cups were placed centrally in the trap and secured to the floor using Blu-Tac^®^ adhesive to elevate the cup above the metaldehyde solution. The experimental design was based on Cordoba et al. (2020) [20]. Traps were tested in pairs, consisting of one treatment and one control, set up in 3 rows of 10 traps (30 traps total, *n* = 15). The placement of each trap within each pair of traps was randomized. The distance between rows was 5 m. The distance between each pair of traps in a row was 2 m, while traps in each pair were separated by 30 cm. All traps were checked 24 h after set-up and the dead gastropods were counted and removed. To test the response of *C. aspersum* and *A. valentianus* to the fermenting dough, an experiment was conducted in a grassy area behind a sand dune in Newport, Oregon (44.623361, −124.049390) 9–10 May 2018. To evaluate the response of *D. reticulatum* to the attractant, the fermenting dough was tested in a white clover (*Trifolium repens*) field in Albany, Oregon (44.391584, −123.125801) 29–30 May 2018.

#### 2.3.2. *Lissachatina fulica* and *Parmarion martensi*

For these trials, a larger trap was designed by cutting four holes into a 35 cm × 20 cm × 12.5 cm Sterilite^®^ 5.7 L plastic container (#1642) to function as suitable entrances for large *L. fulica* and allow for adequate ventilation and dispersal of the bread dough volatiles (Figure S2). Each trap contained 200 mL of liquid metaldehyde (SlugFest^®^) solution at the labelled rate (30 mL in 38 L of water). A 9-cm diameter Petri dish was placed in the approximate center of the trap and secured to the floor using Blu-Tac^®^ adhesive. The Petri dishes elevated the plastic cup containing the bread dough bait or water control above the metaldehyde solution. A 9-cm diameter net pot was placed over each Petri dish to secure and protect the bait/control from possible consumption or removal by rats or other non-target animals. A total of 25 traps were baited with 10 mL of the 2-day old bread dough, and 25 traps with 10 mL of deionized water. Traps were set up just before sunset along the edge of dense brush at two different locations at the Komohana Research & Extension Center in Hilo, Hawaii (19.6961552, −155.0904070). Traps were tested in pairs, consisting of one treatment and one control along the brush edge. The distance between each pair of traps in a row was ~2 m, while traps in each pair were separated by 30 cm. The distance between the two trapping locations was ~150 m. Trapping was performed continuously for 48 h at each location (Location 1: 4–5 June 2018; Location 2: 6–7 June 2018) with gastropods counted and removed every 24 h. The 2-day old dough, water, and metaldehyde were also replaced daily, and the position of traps in each pair was re-randomized. During the final 24 h of the trial at location 2, five pairs of traps were destroyed (possibly by wild pigs) and although dead *L. fulica* and *P. martensi* were visible in the vicinity of the damaged traps it was not possible to assign these specimens to the treatment or control so they were omitted from the data set. 

#### 2.3.3. *Xerolenta obvia*

A mining reclamation site heavily infested with *X. obvia* was selected for field tests with this species. The site, located within Cascade County, Montana (47.382489°, −110.92925°), is owned by the State of Montana, Department of Environmental Quality. At the time of the trial, no snail abatement methods were being used. Attractants and controls were presented to snails as baits in Snailer^®^ traps, or in open plastic Petri dishes (13.5 cm diameter).

##### Snailer^®^ Traps

The same trapping methodology for Snailer^®^ traps was used as described in Section 2.3.1 except that 120 traps total (60 treatment and 60 control) were deployed in six rows of 20 traps (10 pairs). Two 30 mL plastic disposable portion cups (Crystal Ware Clear Portion Cups, Conlon Products, Inc., Lawrence, MA, USA) containing ~15 mL of fermenting bread dough (~30 mL total) were used in the treatment traps, and the same volume of bottled water was used in controls (Figure S3). Trapping was performed continuously for 48 h (18–20 June 2018) with snails counted and removed every 24 h. The 2-day old dough, water, and metaldehyde were replaced daily, at which time the placement of traps in each pair was switched.

##### Petri Dish Bioassays

In addition to testing Snailer^®^ traps, we tested the dough in open Petri dishes, which both provided a larger odor plume than the more enclosed traps, and which avoided the necessity of using relatively expensive commercial traps. The Petri dish bioassays were conducted at the same location but ~50 m from the trapping study. Thirty pairs of Petri dishes (13.5 cm diameter) were deployed in six rows of five pairs of dishes. About 30 mL of dough was placed in a single 60 mL plastic disposable portion cup (Crystal Ware Clear Portion Cups, Conlon Products, Inc., Lawrence, MA, USA) that was secured to the center of the dish using Blu-Tac^®^ adhesive, and 30 mL of liquid metaldehyde (SlugFest^®^) solution at the labelled rate was poured into each dish so that it covered the bottom (Figure S4). The same volume of bottled water was used for the control traps. This trial was run continuously for 48 h (20–22 June 2018). After 24 h the snails were counted and removed, the 2-day old dough, water, and liquid metaldehyde were replaced, and the positions of dishes in each pair were switched.

### 2.4. Effect of Aging on Dough Performance in the Field

The attractiveness of 2-day old dough to *D. reticulatum* in the field was compared with fresh, 1-day old, 4-day old, 6-day old, 8-day old, and 10-day old dough during November 2020 using the methods described in Section 2.3.1 above. Traps were placed in triplicates, consisting of two treatments (2-day old dough and one of the other aged dough treatments) and a water control, such that for each field experiment there were ten triplicates total arranged in two blocks of 15 traps (30 traps total, *n* = 10; Figure S5). The variously aged doughs were tested separately on different dates in a field of established perennial ryegrass (*Lolium perenne*) infested with *D. reticulatum* in Shedd, Oregon (44.437449, −123.120249). Different parts of the field were used to test the differently aged doughs with a minimum of 50 m between testing locations. On completion of this trial it appeared that the number of slugs collected in the control traps was higher than in other field trials, possibly because of the presence of two dough treatments in close proximity to the controls (all other field trials used a single dough treatment). Thus, as a precaution, the trial was repeated in the same field for the 4-day old dough and 8-day old dough in January 2021 but using increased spacing between traps, i.e., 1 m between each trap in each triplicate, 5 m between triplicates, and 10 m between blocks of traps (Figure S5). 

### 2.5. Statistical Analysis

The Shapiro-Wilk test was used to test the data for normality, and despite transformation attempts (e.g., square root, log_10_, and log_10_ + 1) it was not possible to normalize the data sets. Thus, nonparametric statistics were used. Differences in the numbers of specimens collected in the bread dough and control traps and Petri dishes (*X. obvia* only) were tested using either the Mann Whitney U test (1 dough treatment and control) or the Kruskal–Wallis test (2 dough treatments and control). Post-hoc analysis was carried out using Dunn’s test, incorporating the Bonferroni correction for multiple comparisons. Gastropod counts from the 48 h field trials (*L. fulica*, *P. martensi*, and *X. obvia*) were pooled. Chi square analysis was used for the laboratory choice data. Levels of significance corresponding to *p* < 0.05, *p* < 0.01, and *p* < 0.001 were used throughout. All statistical analyses were carried out using IBM^®^ SPSS^®^ Version 26 [33]. 

## 3. Results

### 3.1. Laboratory Choice Bioassays with Cornu aspersum to Test the Attractiveness of Bread Dough

#### 3.1.1. Attractiveness of Dough of Various Ages

**Figure 1:**
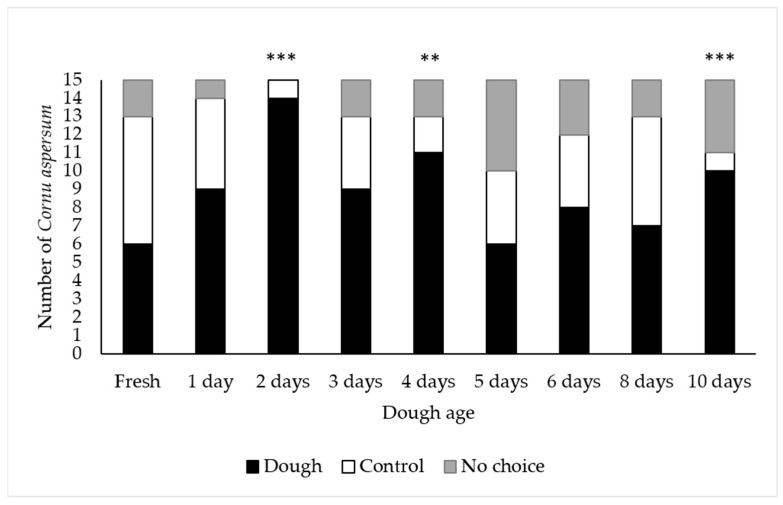
Number of *Cornu aspersum* that selected bread dough, the water control, or that made no choice in laboratory choice tests. Bars with asterisks indicate a significant difference in the number of snails that chose the bread dough versus water control (** = *p* < 0.01; *** = *p* < 0.001). Chi square analysis: Day 1: X^2^ = 0.15, NS; Day 2: X^2^ = 11.33, *p* < 0.001; Day 3: X^2^ = 2.00, NS; Day 4: X^2^ = 6.31, *p* < 0.01; Day 5: X^2^ = 0.50, NS; Day 6: X^2^ = 1.42, NS; Day 8: X^2^ = 0.61, NS; Day 10: X^2^ = 7.44, *p* < 0.001.

Significantly more adult *C. aspersum* selected the bread dough when it was aged for 2 days (*p* < 0.001), 4 days (*p* < 0.01), and 10 days (*p* < 0.001) compared to the water controls (Figure 1). Given that all but one snail selected the 2-day old bread dough, dough aged for 48 h was used for all subsequent laboratory and field trials.

#### 3.1.2. Attractiveness of Dough Aged for 2 Days versus Deadline^®^ M-Ps™

**Figure 2:**
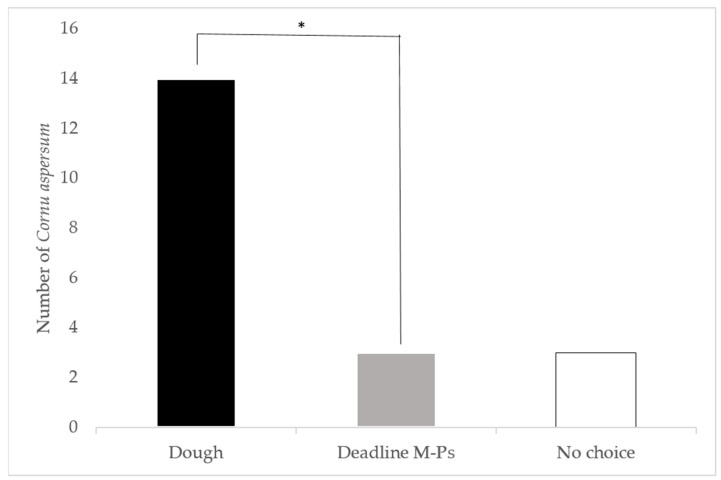
Direct comparison of the attraction of *Cornu aspersum* to two day old bread dough and Deadline^®^ M-Ps™ in laboratory choice trials. *: Snails significantly preferred the dough over the metaldehyde bait (X^2^ = 5.88, *p* < 0.05).

In a direct comparison of 2-day old dough versus the commercial Deadline^®^ M-Ps™ bait, 14 snails selected the dough, three selected the commercial bait, and three were classed as non-responders (Figure 2). Significantly more *C. aspersum* selected the two dayold dough than the Deadline^®^ M-Ps™ (*p* < 0.05).

### 3.2. Field Trials with Dough Aged for 2 Days

#### 3.2.1. *Cornu aspersum* and *Ambigolimax valentianus*

**Figure 3:**
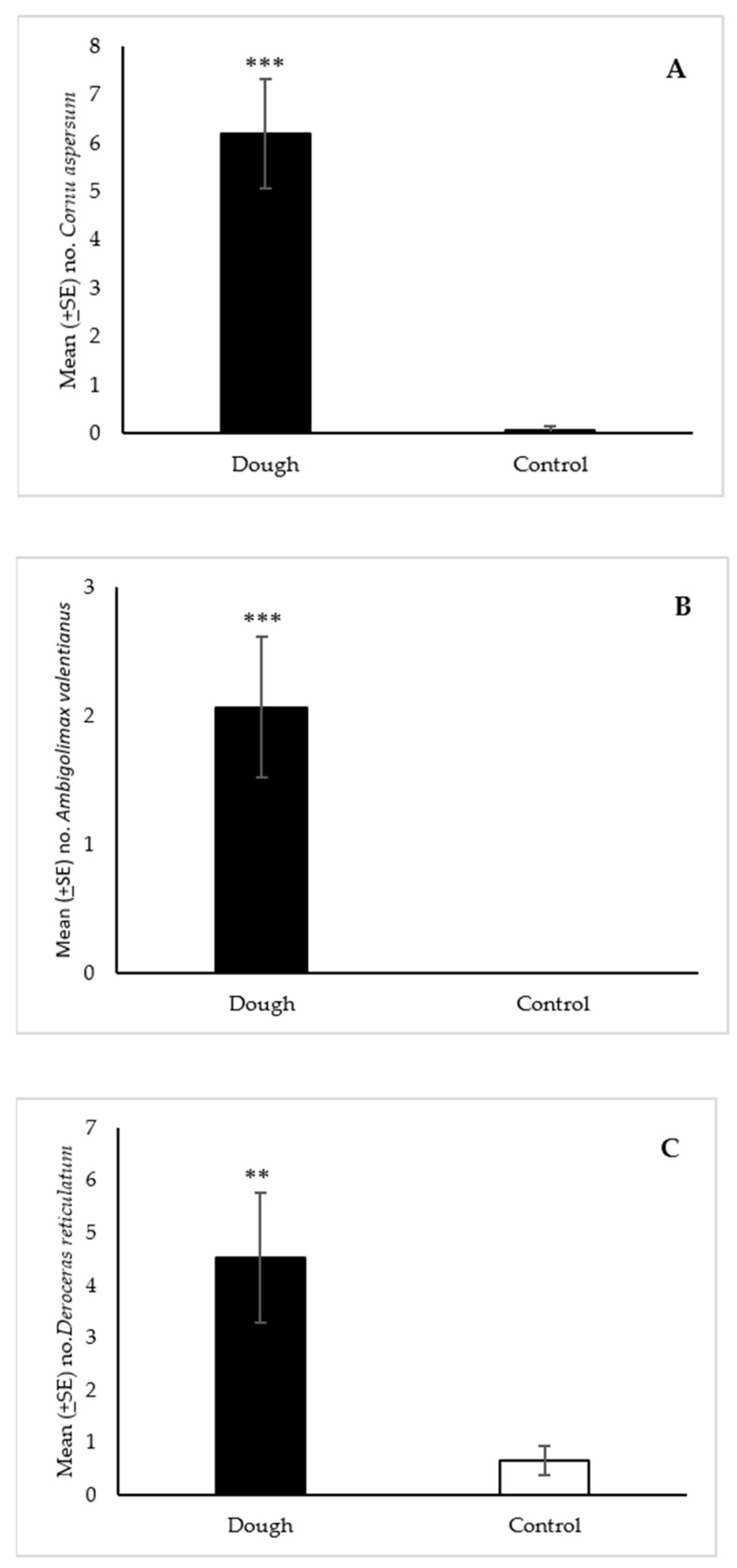
Mean (±SE) number of *Cornu aspersum* (**A**), *Ambigolimax valentianus* (**B**), and *Deroceras reticulatum* (**C**) collected per trap over 24 h in Snailer^®^ traps baited with 2-day aged bread dough or a water control in field trials in Oregon. Traps also contained liquid metaldehyde at labeled rate. Treatment bars with asterisks indicate significant differences (** *p* < 0.01, *** *p* < 0.001) between the median number of specimens collected in the dough and control traps. Mann Whitney U test (n = 15): (**A**) standardized test statistic = 4.60, *p* < 0.001; (**B**) standardized test statistic = 3.70, *p* < 0.001; (**C**) standardized test statistic = 3.29, *p* < 0.01.

For *C. aspersum*, a total of 93 snails were collected in the dough-baited traps compared to 1 snail in the controls (Figure 3A). The statistical analysis showed that the median number of *C. aspersum* (6) collected with the dough was significantly greater (*p* < 0.001) than the water control (0). For *A. valentianus*, 31 slugs were collected in total in the dough-baited traps while no slugs were collected in the controls. Prior to testing the dough, we were unaware of the presence of *A. valentianus* at this site. The median number of slugs collected with the dough (1) was significantly greater (*p* < 0.001) than the water control (0) (Figure 3B).

#### 3.2.2. *Deroceras reticulatum*

A total of 68 and 10 slugs were collected with the dough and water controls respectively. An analysis of medians showed that significantly more (*p* < 0.01) *D. reticulatum* were collected in the dough-baited traps (3) than in the controls (0) (Figure 3C).

#### 3.2.3. *Lissachatina fulica* and *Parmarion martensi*

**Figure 4:**
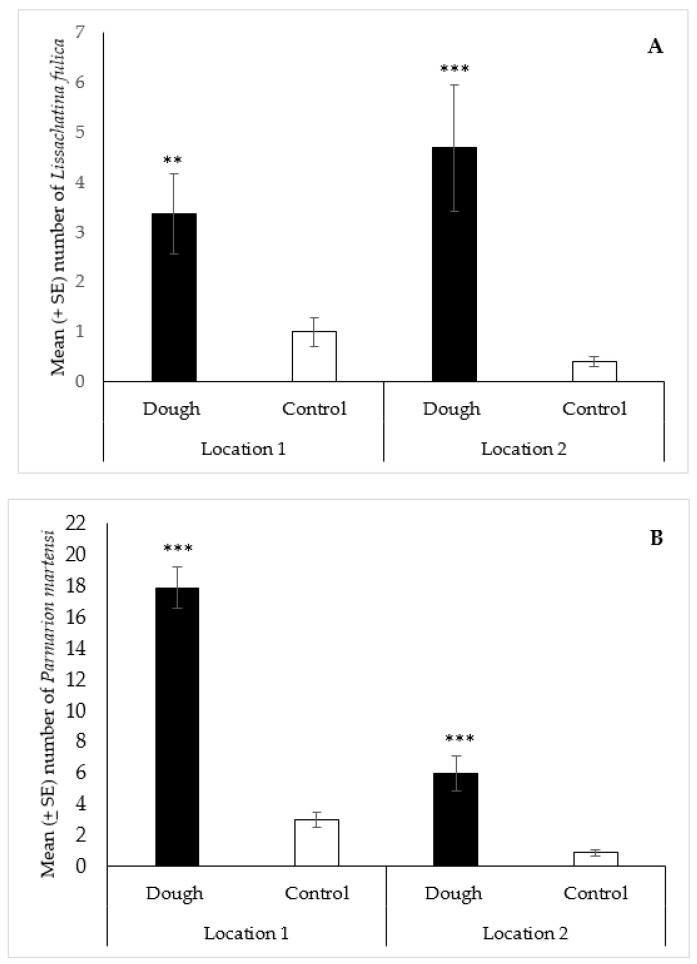
Mean (±SE) number of *Lissachatina fulica* (**A**) and *Parmarion martensi* (**B**) collected per trap over 48 h in traps baited with 2-day aged bread dough or a water control, in field trials in two locations in Hilo, Hawaii. Traps also contained liquid metaldehyde at labeled rate. Treatment bars with asterisks indicate significant differences (** *p* < 0.01, *** *p* < 0.001) between the median number of specimens collected in the dough and control traps. Mann Whitney U test (n = 25): (**A**) location 1—standardized test statistic = 3.22, *p* < 0.01; location 2—standardized test statistics = 3.49, *p* < 0.001; (**B**) location 1—standardized test statistics = 6.02, *p* < 0.001; location 2—standardized test statistic = 4.42, *p* < 0.001.

For *L. fulica*, a total of 201 specimens (84 at location 1; 117 at location 2) were collected with the dough over the course of the study, compared to 35 in the controls (25 at location 1; 10 at location 2). The statistical analysis showed that the median number of *L. fulica* (2) collected with the dough was significantly greater (*p* < 0.01) than the water control (1) at location 1 (Figure 4A). Similarly, at location 2, the median number of *L. fulica* (3) collected with the dough was significantly greater (*p* < 0.001) than the control (0) (Figure 4A). For *P. martensi*, 595 specimens (446 at location 1; 149 at location 2) were collected with the dough bait during the field trial, compared to 97 in the controls (75 at location 1; 22 at location 2). The median number of *P. martensi* (18) collected with the dough was significantly greater (*p* < 0.001) than the water control (2) at location 1 (Figure 4B). Similarly, at location 2, the median number of *P. martensi* (3) collected with the dough was significantly greater (*p* < 0.001) than the control (1) (Figure 4B).

#### 3.2.4. *Xerolenta obvia*

##### Snailer^®^ Traps

**Figure 5:**
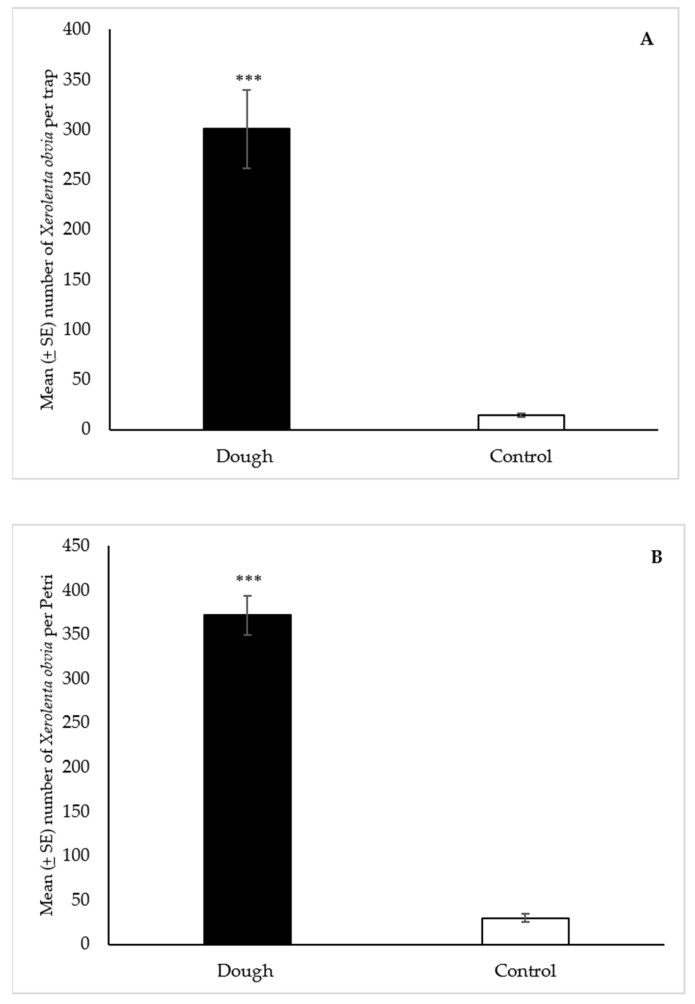
Mean (±SE) number of *Xerolenta obvia* collected per trap over 48 h in Snailer^®^ traps (**A**) and Petri dishes (**B**) baited with 2-day aged bread dough or a water control, in field trials near Belt, Montana. Traps also contained liquid metaldehyde at labeled rate. Treatment bars with asterisks indicate significant differences (*** *p* < 0.001) between the median number of specimens collected in the dough and control traps. Mann Whitney U test: (**A**) standardized test statistic = 7.08, n = 60, *p* < 0.001; (**B**) standardized test statistic = 6.65, n = 30, *p* < 0.001.

A total of 18,049 *X. obvia* were collected in the dough-baited Snailer^®^ traps over the course of the study, compared to 864 in the controls. The statistical analysis showed that the median number of snails (166) collected with the dough was significantly greater (*p* < 0.001) than the water control (10) (Figure 5A).

##### Petri Dishes

A total of 11,154 *X. obvia* were collected in the dough-baited Petri dishes, compared to 893 in the controls. The statistical analysis showed that the median number of snails (355) collected with the dough was significantly greater (*p* < 0.001) than the water control (22) (Figure 5B).

### 3.3. Effect of Aging on Dough Performance in the Field

**Figure 6:**
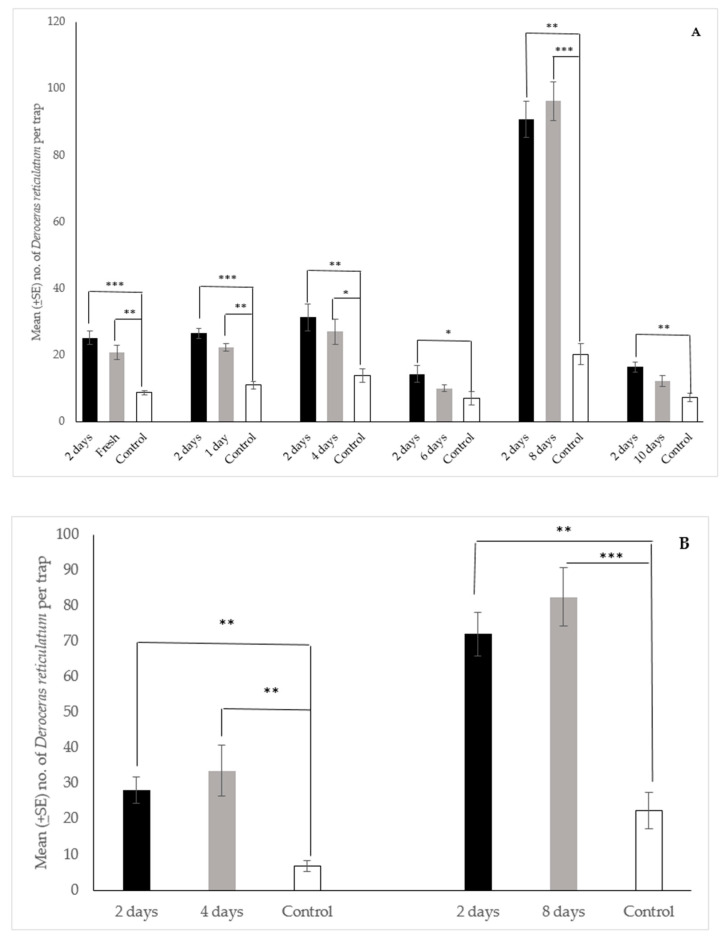
Mean (±SE) number of *Deroceras reticulatum* collected per Snailer^®^ trap over 24 h with original spacing i.e., 30 cm (**A**) and increased spacing i.e., 1 m (**B**) between traps. Traps baited with 2- day aged dough, dough of various ages, or a water control in field trials in Oregon. Traps also contained liquid metaldehyde at labeled rate. Kruskal-Wallis Test (n = 10, df = 2), pairwise post-hoc tests with significance values adjusted by the Bonferroni correction for multiple tests. Bars with asterisks indicate significant differences (* *p* < 0.05, ** *p* < 0.01, *** *p* < 0.001) between the median values. (**A**). Original trap spacing: Fresh dough—H = 18.17, *p* = 0.000; 2 day vs. control, test statistic = 4.09, *p* < 0.001; fresh vs. control, test statistic = 3.09, *p* < 0.01; 1-day old dough—H = 20.56, *p* = 0.000; 2 day vs. control, test statistic = 4.44, *p* < 0.001; 1 day vs. control, test statistic = 3.01, *p* < 0.01; 4-day old dough—H = 11.92, *p* = 0.003; 2 day vs. control, test statistic = 3.31, *p* < 0.01; 4 day vs. control, test statistic = 2.50, *p* < 0.05; 6-day old dough—H = 8.15, *p* = 0.017; 2 day vs. control, test statistic = 2.81, *p* < 0.05; 8-day old dough—H = 19.42, *p* = 0.000; 2 day vs. control, test statistic = 3.70, *p* < 0.01; 8 day vs. control, test statistic = 3.93, *p* < 0.001; 10-day old dough—H = 14.19, *p* = 0.001; 2 day vs. control, test statistic = 3.73, *p* < 0.01. (**B**). Increased trap spacing: 4-day old dough—H = 16.94, *p* = 0.000; 2 day vs. control, test statistic = 3.58, *p* < 0.01; 4 day vs. control, test statistic = 3.55, *p* < 0.01; 8-day old dough—H = 18.61, *p* = 0.000; 2 day vs. control, test statistic = 3.49, *p* < 0.01; 8 day vs. control, test statistic = 3.94, *p* < 0.001.

The data from the field trials using a spacing of 30 cm between traps in each triplicate are presented in Figure 6A. In all cases there were no statistical differences in the median number of slugs collected in traps between the two-day old dough and dough that was fresh, 1-day old, 4-days old, 6-days old, 8-days old, and 10-days old. In addition, traps baited with freshly prepared dough, or dough aged for 1 day, 4 days, or 8 days collected significantly more slugs than the control traps, while traps baited with the 6 day and 10-day old dough did not. Similarly, the median number of slugs collected in traps baited with the 2-day old dough was significantly greater than the control traps for each trial (Figure 6A).

Likewise, in the trial using an increased spacing of 1 m between traps in each triplicate (Figure 6B) there were no statistical differences in the median number of slugs collected in traps between the two-day old dough and dough that was aged for 4 days and 8 days. However, traps baited with the 2, 4, and 8-days old dough collected significantly more slugs than control traps (Figure 6B). In sum, the data from both data sets indicate that the dough has an active field lifetime of at least 8 days, and becomes attractive essentially as soon as it is mixed.

## 4. Discussion

Although beer has long been known to attract gastropods, other fermenting products likely have potential to be used as attractants as well. For example, the Internet is rich with anecdotes and recipes for homemade fermenting attractants for the home gardener (e.g., SlugChug—https://www.theartofdoingstuff.com/diy-slug-bait/ (accessed on 15 March 2021)). However, to the best of our knowledge these types of formulations have not been tested in robust quantitative field trials. This knowledge gap provided the incentive for this study. The resulting data supported our hypothesis that very simply prepared fermenting bread dough was highly attractive to six key invasive gastropod species at infested locations in Hawaii, Oregon, and Montana. The native ranges of these six species encompass Central and Western Europe [34,35], East Africa [36], and Southeast Asia [37], highlighting the generic attractiveness and potential usefulness of bread dough baits in invasive gastropod management worldwide. 

Over the past century, invasive populations of *L. fulica* have been eradicated in the U.S. (e.g., California), Australia (e.g., Queensland), and some of the Pacific Islands (e.g., Fiji and Western Samoa), often at great expense [36]. There is an ongoing, costly eradication program in Miami, Florida [19], attempting to stamp out a population that became established around 2011, and it is very probable that *L. fulica* will continue to invade new regions of the world as global trade and tourism increase. The problem is exacerbated by the intentional introduction and rearing of *L. fulica* for consumption, as pets [38], and for use in religious rituals [39]. Based on our data, fermenting bread dough should be considered as a valuable new tool to help detect and eradicate future and current incipient invasive populations of this species. Given that eosinophilic meningoencephalitis vectored by *L. fulica* [29] is regarded as a globally emerging infectious disease [40], the development of tools to help manage its gastropod vectors is of the utmost importance. The potential for another of our target species, *P. martensi*, to transmit this disease is likely higher than other gastropod species because of the high prevalence of infection, high nematode loads, and its greater association with homes, increasing the possibility of human–mollusk interactions [30,41]. In Hawaii, where *P. martensi* is a relatively recent introduction, control measures rely on sanitation and baits, but additional strategies are urgently needed [17] given how prevalent the semislug has become, and the corresponding increased prevalence of the disease in humans [40]. Use of bread dough-based baits for detection and control could represent a simple strategy to help meet this management need. 

Bread dough lures also have major potential for managing or possibly eradicating populations of our other target species. For example, eradication efforts over several years have failed to eliminate established populations of *X. obvia* in Montana and Michigan [35] and new tools are clearly needed. In particular, these populations still appear to be limited in range, and so eradication may still be possible with an effective bait. 

Other target species are so widely distributed that eradication is not an option. Nevertheless, an effective attractant can still be a valuable addition to the arsenal of tools available to manage these pests. For example, *C. aspersum* is a key quarantine pest of the nursery industry on the west coast of the United States [42], and dough baits could be used to ensure that holding areas in nurseries are snail free before shipping to other states where the snail is not known to be present, such as Florida [43]. The same is true for *A. valentianus* and *D. reticulatum*, which are also important pests of ornamentals and other crops [25,44]. For all of these species, our data suggest that bread dough will likely be useful as a simple and economical attractant to enhance detection, mass trapping, and attract-and-kill strategies for managing these invasive pests. Dough-baited traps could also be used as an early detection tool at high-risk locations such as airports and seaports, and particularly, container yards and warehouses where shipments are opened for redistribution across the country. Effective new lures could be particularly useful in vulnerable areas (e.g., for *X. obvia* in Oregon, *C. aspersum* in Florida, and *P. martensi* on the Gulf Coast) to detect new populations as soon as possible after they arrive, and hence ultimately reduce eradication costs. This role would be facilitated by the fact that the dough appears to have an active field life of at least 8 days and consequently costs associated with lure replacement (e.g., labor, supplies) will be minimized. However, it must be pointed out that if baited traps are left out for extended periods of a week or more without removing the trapped/dead gastropods, the odor from their decay may influence the attraction of additional slugs or snails to the baited traps.

Although identifying the active components in the dough and reconstructing an attractive blend was not an aim of this study, it may be a fruitful avenue for future research. The odor profile of fermenting bread dough has been the subject of past research (e.g., [45,46]) and these studies would be a useful starting point for the identification of the active compounds. The ethanol produced during fermentation does not appear to be responsible for the observed attraction of gastropods to beer and other fermenting products because previous research ([47]) demonstrated that for *D. reticulatum*, an alcohol-free malt beverage attracted more slugs than similar beverages containing alcohol, and that alcohol fortification of beers had a variable and often negative effect. Cranshaw [47] also showed that sugar, water, and yeast combinations were highly attractive, as was malted grain fiber (brewery waste product) amended with sucrose and active yeasts. 

Nevertheless, regardless of the active compounds, bread dough constitutes a cheap, and effective option for managing gastropod infestations. In fact, given the simplicity and the fact that the low cost ingredients are available almost anywhere in the world, bread dough could be used as a pest gastropod management tool in developing countries where access to commercial molluscicide baits is limited by cost. For example, one of our target species, *L. fulica*, causes significant crop damage throughout Africa and Asia [36], and it has recently invaded many islands in the Caribbean [48]. Furthermore, it would be very straightforward to formulate and package a cheap, dry commercial formulation of flour and yeast where the applicator simply adds water to activate the bait. Such a formulation would likely have an indefinite shelf life and be readily shippable, both highly desirable properties for an operational lure. In areas of the world where molluscicides are the mainstay of pest gastropod control, bread dough presents an alternative attractant that could be incorporated into baits to increase their efficacy. Our data, for example, showed that dough aged for 2 days was significantly more attractive than a widely used metaldehyde bait to the global invasive pest, *D. reticulatum*.

In conclusion, given that fermenting bread dough is attractive to at least six species (*D. reticulatum*, *C. aspersum*, *A. valentianus*, *L. fulica*, *P. martensi*, and *X. obvia*) of gastropods, which includes examples of snails, slugs, and semislugs from both temperate and tropical areas of the world, we suggest that it also be tested as a possible attractant for other key pest mollusk taxa both in the U.S. (e.g., *Theba pisana* in California; *Macrochlamys indica* in Florida) and other regions (e.g., *Cochlicella barbara* and *C. acuta* in Australia; *Arion vulgaris* in Europe).

## Data Availability

The data presented in this study are available on request from the corresponding author.

## Supplementary Materials

The following are available online at https://www.mdpi.com/article/10.3390/insects12040328/s1. Figure S1: Snailer^®^ trap. Figure S2: Trap baited with fermenting bread dough (upper) and water control (lower) from field trials with *Lissachatina fulica* in Hawaii. Figure S3: Snailer^®^ trap baited with fermenting bread dough (right) and water control (left) from field trials with *Xerolenta obvia* in Montana. Figure S4: Petri dish baited with fermenting bread dough (left) and water control (right) from field trials with *Xerolenta obvia* in Montana. Figure S5: Experimental design for assessing effect of aging on dough performance in the field.

## References

[1] Barker G.M. (2002). Molluscs as Crop Pests.

[2] Cowie R.H. (2001). Can snails ever be effective and safe biocontrol agents?. Int. J. Pest Manag..

[3] Bailey S.E.R., Barker G.M. (2002). Molluscicidal baits for control of terrestrial gastropods. Molluscs as Crop Pests.

[4] Hammond R.B., Byers R.A., Barker G.M. (2002). Agriolimacidae and Arionidae as pests in conservation tillage soybean and maize cropping in North America. Molluscs as Crop Pests.

[5] Henderson I., Triebskorn R., Barker G.M. (2002). Chemical control of terrestrial gastropods. Molluscs as Crop Pests.

[6] Godan D. (1983). Pest Slugs and Snails: Biology and Control.

[7] Barker G.M., Pottinger R.P., Lloyd J.M., Addison P.J., Firth A.C., Stewart A.P. (1991). A Novel Bait Formulation for Slug and Snail Control.

[8] Rae R., Verdun C., Grewal P.S., Robertson J.F., Wilson M.J. (2007). Biological control of terrestrial molluscs using *Phasmarhabditis hermaphrodita*—progress and prospects. Pest Manag. Sci..

[9] Sakovich N.J., Barker G.M. (2002). Integrated management of *Cantareus aspersus* (Müller) (Helicidae) as a pest of citrus in California. Molluscs as Crop Pests.

[10] Hammond R.B., Stinner B.R. (1987). Seedcorn maggots (Diptera: Anthomyiidae) and slugs in conservation tillage systems in Ohio. J. Econ. Entomol..

[11] Hirano T., Yamazaki D., Uchida S., Saito T., Chiba S. (2019). First record of the slug species *Semperula wallacei* (Issel, 1874) (Gastropoda: Eupulmonata: Veronicellidae) in Japan. BioInvasions Rec..

[12] Serniotti E.N., Guzmán L.B., Beltramino A.A., Vogler R.E., Rumi A., Peso J.G. (2019). New distributional records of the exotic land snail *Bradybaena similaris* (Férussac, 1822) (Gastropoda, Bradybaenidae) in Argentina. BioInvasions Rec..

[13] Vendetti J.E., Burnett E., Carlton L., Curran A.T., Lee C., Matsumoto R., Donnell R.J., Reich I., Willadsen O. (2019). The introduced terrestrial slugs *Ambigolimax nyctelius* (Bourguignat, 1861) and *Ambigolimax valentianus* (Férussac, 1821) (Gastropoda: Limacidae) in California, with a discussion of taxonomy, systematics, and discovery by citizen science. J. Nat. Hist..

[14] Mc Donnell R.J., Vlach J.J., Reich I., Colton A.J. (2020). *Boettgerilla pallens* Simroth, 1912 (Boettgerillidae): A new invasive slug species in Oregon, U.S.A. Am. Malacol. Bull..

[15] Horsák M., Naggs F., Backeljau T. (2020). *Paropeas achatinaceum* (Pfeiffer, 1846) and other alien subulinine and opeatine land snails in European greenhouses (Gastropoda, Achatinidae). Malacologia.

[16] El-Danasoury H., Iglesias-Piñeiro J., Córdoba M. (2016). The effect of climate manipulations on the herbivory of the pest slug *Deroceras reticulatum* (Müller, 1774) (Pulmonata: Agriolimacidae). Int. J. Biometeorol. Heidelb..

[17] Hollingsworth R.G., Howe K., Jarvi S.I. (2013). Control measures for slug and snail hosts of *Angiostrongylus cantonensis*, with special reference to the semi-slug *Parmarion martensi*. Hawaii J. Med. Public Health.

[18] Cordoba M., Millar J.G., Mc Donnell R. (2018). Development of a high-throughput laboratory bioassay for testing potential attractants for terrestrial snails and slugs. J. Econ. Entomol..

[19] Roda A., Millar J.G., Jacobsen C., Veasey R., Fujimoto L., Hara A., McDonnell R.J. (2019). A new synthetic lure for management of the invasive giant African snail, *Lissachatina fulica*. PLoS ONE.

[20] Cordoba M., Millar J.G., Foley I., Anderson T.O., Roda A.L., Adams G.D., Donnell R.J. (2020). Fresh cucumber as an attractant for the invasive snail *Xerolenta obvia*. Am. Malacol. Bull..

[21] Lucid M.K., Ehlers S., Robinson L., Cushman S.A. (2018). Beer, brains, and brawn as tools to describe terrestrial gastropod species richness on a montane landscape. Ecosphere.

[22] Piechowicz B., Grodzicki P., Ząbkiewicz P., Sobczyk A., Dąbrowska A., Piechowicz I., Pieniążek M., Balawejder M., Zareba L. (2018). Components of the smell of beer as enticing factor for invasive slugs *Arion lusitanicus* Non-Mabille. Ecol. Chem. Eng. A.

[23] Pol J., Gries R., Gries G. (2018). Rye Bread and synthetic bread odorants—Effective trap bait and lure for German cockroaches. Entomol. Exp. Appl..

[24] Rowson B., Turner J., Anderson R., Symondson B. (2014). Slugs of Britain and Ireland: Identification, Understanding and Control.

[25] Jeong K.J., Lee S.W., Hong J.K., Shin C.Y., Yun J.G. (2012). Effective control of slug damage through tobacco extract and caffeine solution in combination with alcohol. Hortic. Environ. Biotechnol..

[26] Sanderson G., Sirgel W., Barker G.M. (2002). Helicidae as pests in Australian and South African grapevines. Molluscs as Crop Pests.

[27] Port G., Ester A., Barker G.M. (2002). Gastropods as pests in vegetable and ornamental crops in Western Europe. Molluscs as Crop Pests.

[28] Invasive Species Specialist Group ISSG (2020). The Global Invasive Species Database. http://www.iucngisd.org/gisd/100_worst.php.

[29] Lo Re V., Gluckman S.J. (2003). Eosinophilic meningitis. Am. J. Med..

[30] Hollingsworth R.G., Kaneta R., Sullivan J.J., Bishop H.S., Qvarnstrom Y., da Silva A.J., Robinson D.G. (2007). Distribution of *Parmarion cf. martensi* (Pulmonata: Helicarionidae), a new semi-slug pest on Hawai‘i Island, and its potential as a vector for human angiostrongyliasis. Pac. Sci..

[31] Brodie G., Barker G. (2012). Parmarion martensi Simroth, 1893. Family Ariophantidae.

[32] Speiser B., Barker G.M. (2001). Food and feeding behaviour. The Biology of Terrestrial Molluscs.

[33] IBM Corp (2016). IBM SPSS Statistics for Windows, Version 24.0.

[34] Mc Donnell R., Paine T., Gormally M. (2009). Slugs: A Guide to the Native and Invasive Fauna of California.

[35] Forsyth R.G., Oldham M.J., Snyder E., Schueler F.W., Layberry R. (2015). Forty Years Later: Distribution of the introduced heath snail, *Xerolenta obvia*, in Ontario, Canada (Mollusca: Gastropoda: Hygromiidae). Check List.

[36] Raut S.K., Barker G.M., Barker G.M. (2002). Achatina fulica Bowdich and other Achatinidae as pests in tropical agriculture. Molluscs as Crop Pests.

[37] Cowie R., Hayes K., Kim J., Bustamente K., Yeung N. (2018). *Parmarion martensi* Simroth, 1893 (Gastropoda: Ariophantidae), an intermediate host of Angiostrongylus cantonensis (rat lungworm), on Maui. Bish. Mus. Occas. Pap..

[38] USDA APHIS Giant African Snail. https://www.aphis.usda.gov/aphis/resources/pests-diseases/hungry-pests/the-threat/giant-african-snail/giant-african-snail.

[39] Léo Neto N.A., Voeks R.A., Dias T.L., Alves R.R. (2012). Mollusks of Candomblé: Symbolic and ritualistic importance. J. Ethnobiol. Ethnomedicine.

[40] Cowie R.H. (2017). *Angiostrongylus cantonensis:* Agent of a sometimes fatal globally emerging infectious disease (rat lungworm disease). ACS Chem. Neurosci..

[41] Kim J.R., Hayes K.A., Yeung N.W., Cowie R.H. (2014). Diverse gastropod hosts of *Angiostrongylus cantonensis*, the rat lungworm, globally and with a focus on the Hawaiian Islands. PLoS ONE.

[42] Mc Donnell R., Yoo J., Patel K., Rios L., Hollingsworth R., Millar J., Paine T. (2016). Can essential oils be used as novel drench treatments for the eggs and juveniles of the pest snail *Cornu aspersum* in potted plants?. J. Pest Sci..

[43] Dekle G.W., Fasulo T.R. (2017). Featured Creatures. Brown Garden Snail—Cornu asperum (Müller).

[44] Bergey E.A., Figueroa L.L., Mather C.M., Martin R.J., Ray E.J., Kurien J.T., Westrop D.R., Suriyawong P. (2014). Trading in snails: Plant nurseries as transport hubs for non-native species. Biol. Invasions.

[45] Frasse P., Lambert S., Richard-Molard D., Chiron H. (1993). The influence of fermentation on volatile compounds in french bread dough. LWT Food Sci. Technol..

[46] Gassenmeier K., Schieberle P. (1995). Potent aromatic compounds in the crumb of wheat bread (french-type)—Influence of pre-ferments and studies on the formation of key odorants during dough processing. Z. Für Lebensm. Unters. Forsch..

[47] Cranshaw W. (1997). Attractiveness of Beer and Fermentation Products to the Gray Garden Slug, Agriolimax Reticulatum (Muller) (Mollusca: Limacidae).

[48] CABI Invasive Species Compendium. Datasheet, Achatina Fulica (Giant African Land Snail). https://www.cabi.org/isc/datasheet/2640#todistribution.

