# Supplementary material for: Fermenting Bread Dough as a Cheap, Effective, Nontoxic, and Generic Attractant for Pest Snails and Slugs
Source: Insects. 2021 Apr 7;12(4):328. doi: 10.3390/insects12040328 (PMC8067611; doi:10.3390/insects12040328)
Supplement: Supplementary file 1 [file insects-12-00328-s001.zip › Figure S5 - Experimental design for assessing effect of aging on dough performance in the field.pptx]

## Slide 1
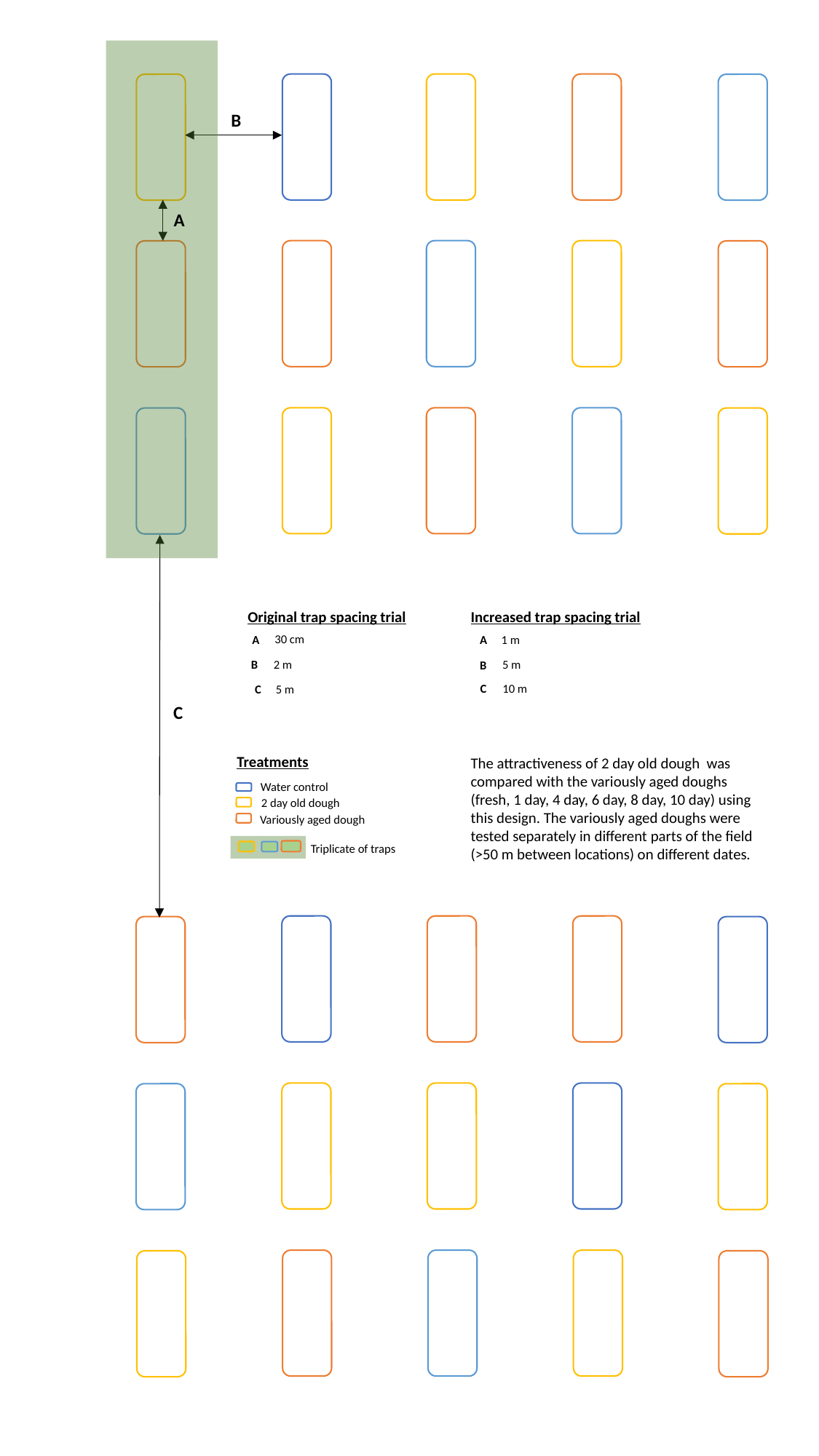

B
A
Increased trap spacing trial
Original trap spacing trial
30 cm
A
1 m
A
B
2 m
5 m
B
C
10 m
C
5 m
C
Treatments
The attractiveness of 2 day old dough was compared with the variously aged doughs (fresh, 1 day, 4 day, 6 day, 8 day, 10 day) using this design. The variously aged doughs were tested separately in different parts of the field (>50 m between locations) on different dates.
Water control
2 day old dough
Variously aged dough
Triplicate of traps
