# Supplementary figures and images for: Fermenting Bread Dough as a Cheap, Effective, Nontoxic, and Generic Attractant for Pest Snails and Slugs
Source: Insects. 2021 Apr 7;12(4):328. doi: 10.3390/insects12040328 (PMC8067611; doi:10.3390/insects12040328)

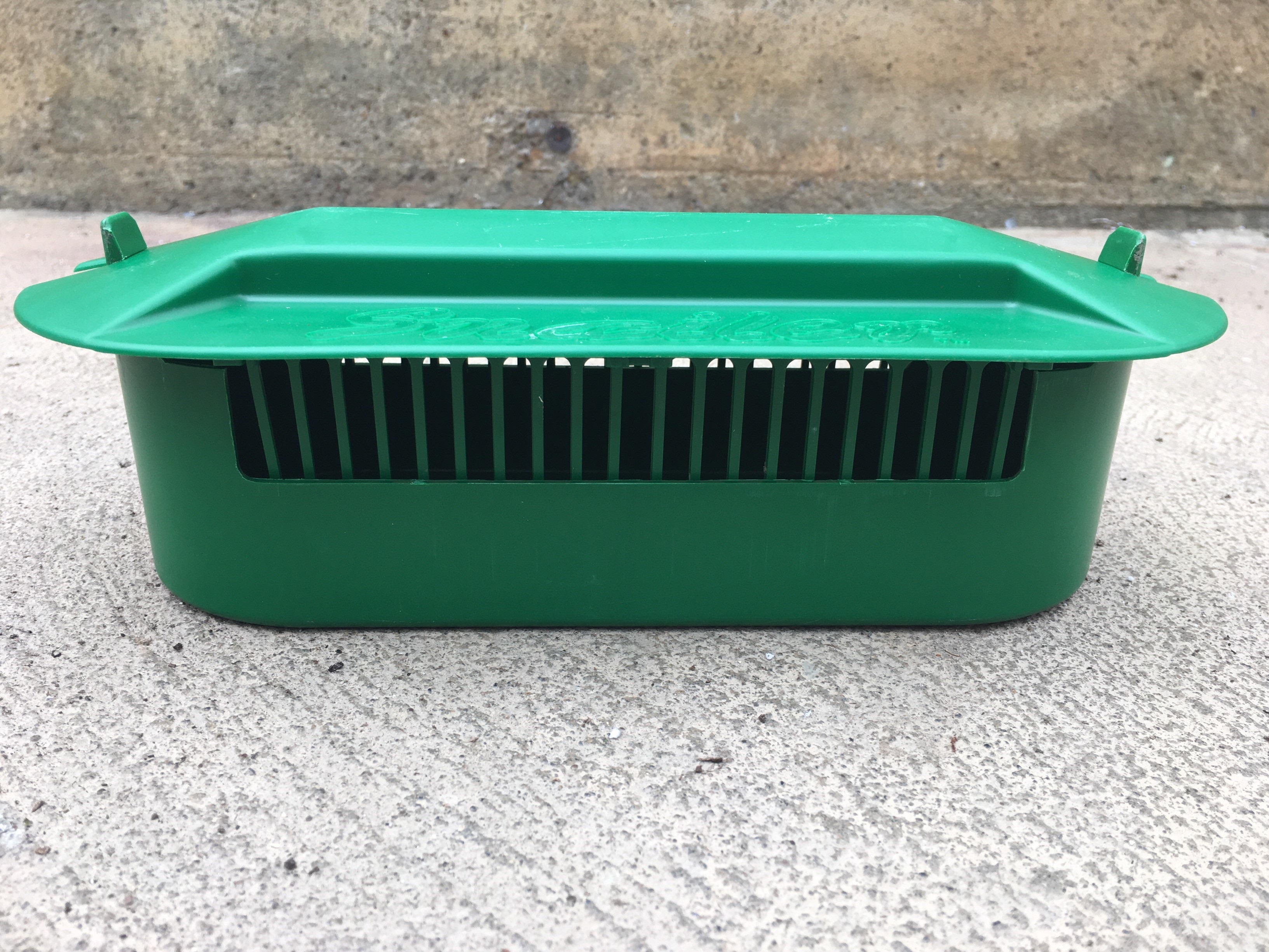

Supplement: Supplementary file 1 [file insects-12-00328-s001.zip › Figure S1 - Snailer trap.jpg]

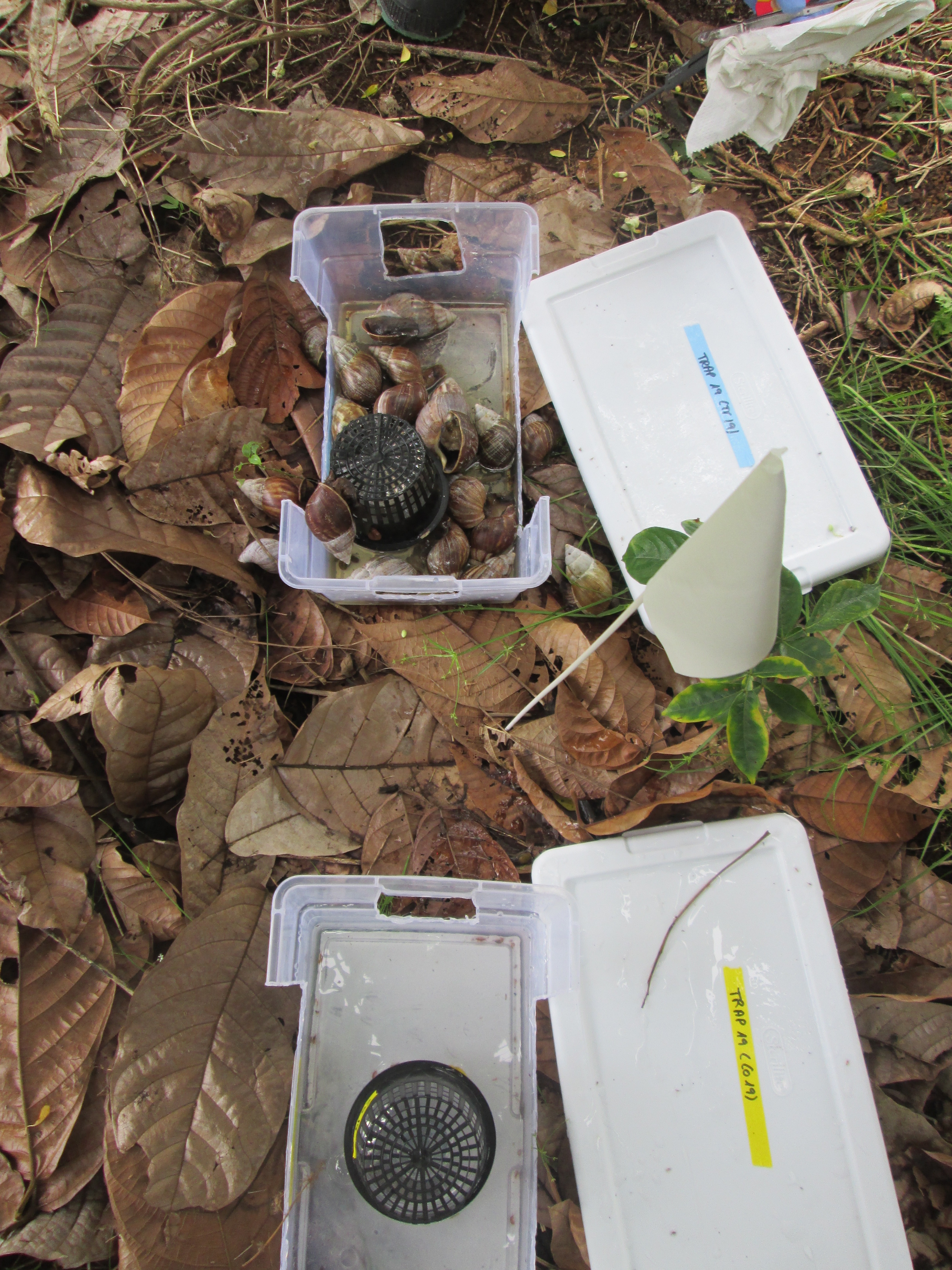

Supplement: Supplementary file 1 [file insects-12-00328-s001.zip › Figure S2-Trap baited with fermenting bread dough (upper) and water control (lower) from field trials in Hawaii.jpg]

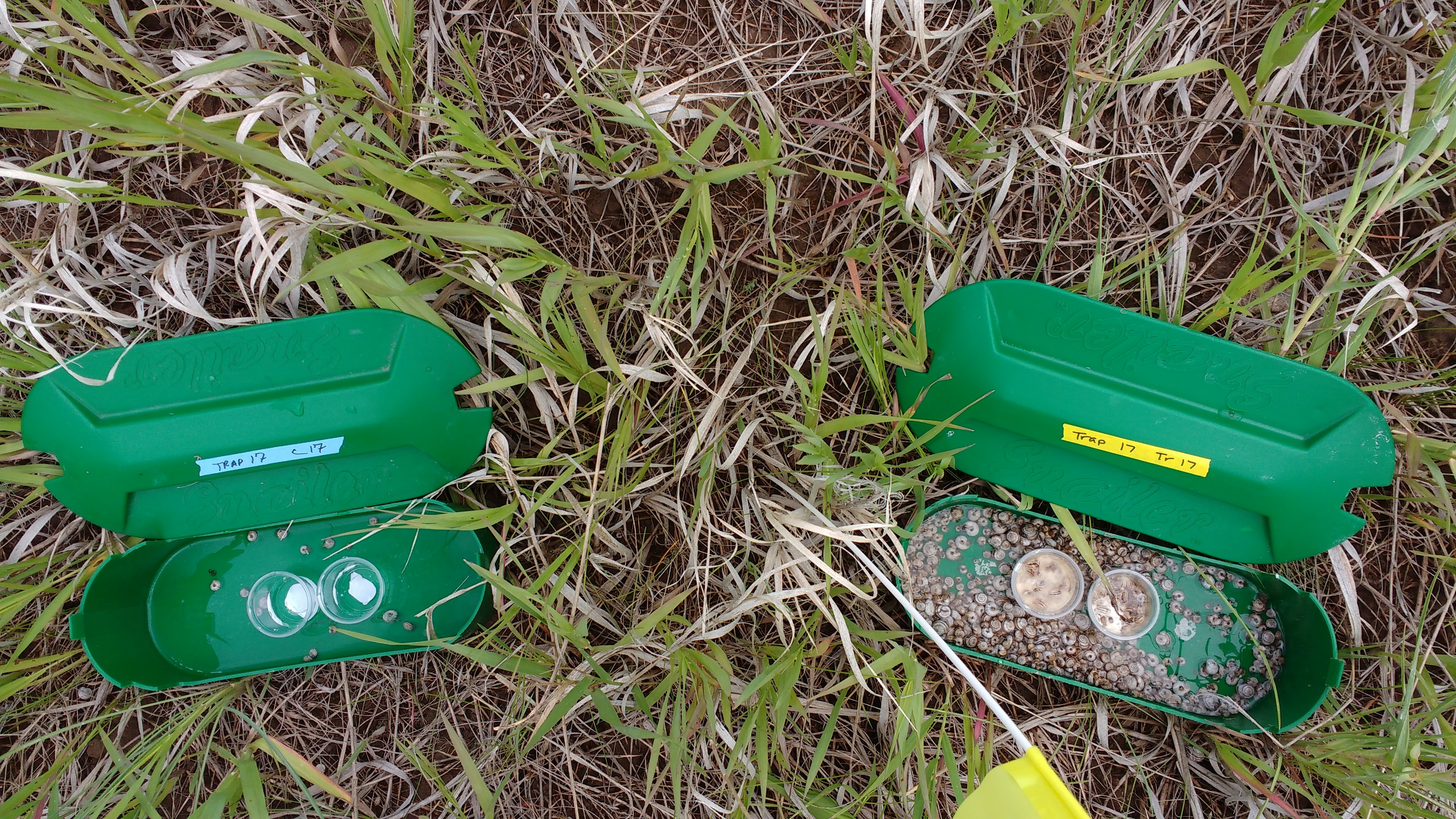

Supplement: Supplementary file 1 [file insects-12-00328-s001.zip › Figure S3-Snailer trap baited with fermenting bread (right) dough and water control (left) from field trials in Montana.jpg]

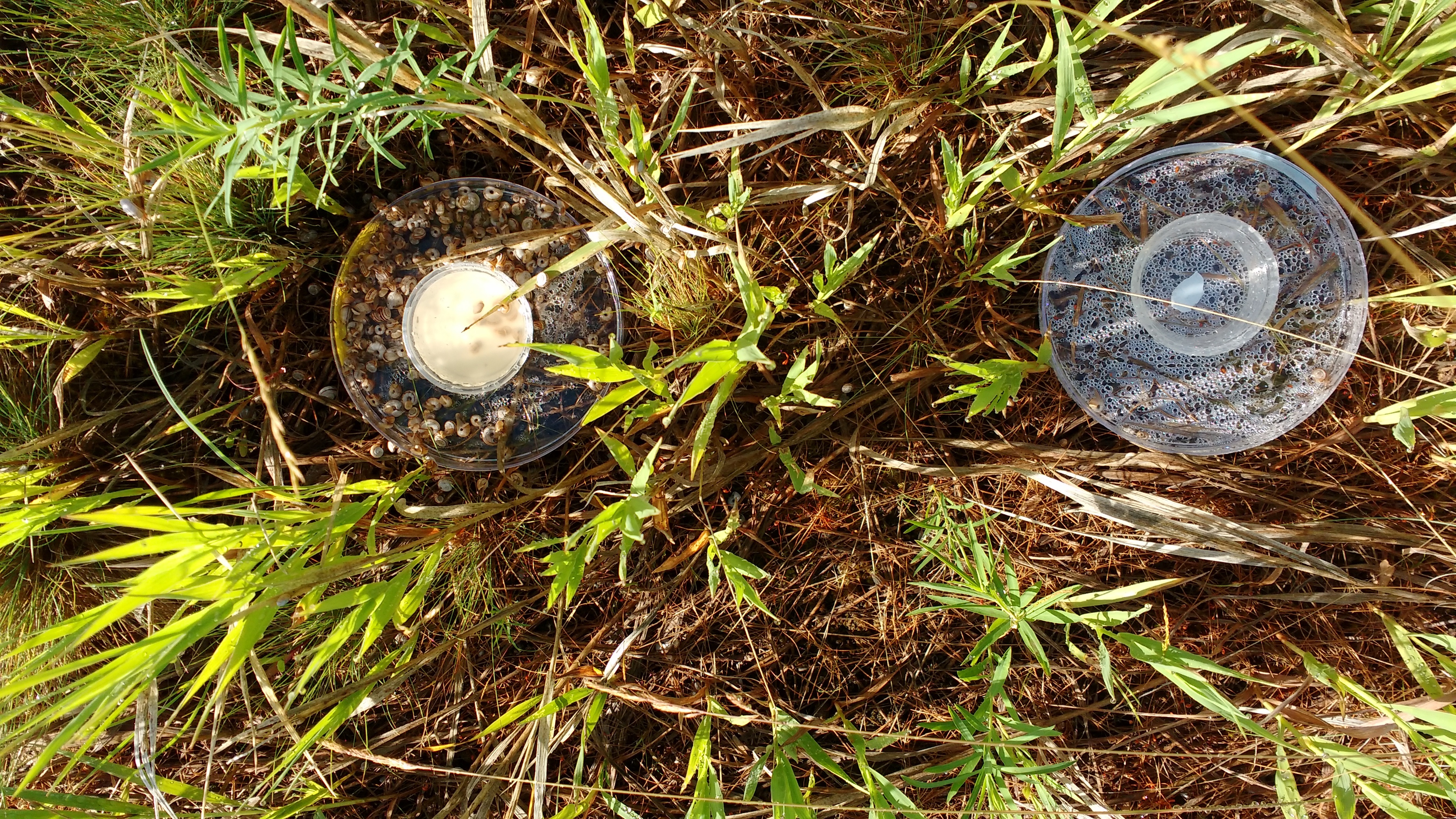

Supplement: Supplementary file 1 [file insects-12-00328-s001.zip › Figure S4 - Petri dish baited with fermenting bread dough (left) and water control (right) from field trials in Montana.jpg]
